# The 2025 Nobel Prize in Physiology or Medicine — a bridge to peripheral immune tolerance

**DOI:** 10.1172/JCI202216

**Published:** 2025-12-01

**Authors:** Jeffrey A. Bluestone

**Affiliations:** Sonoma Biotherapeutics, South San Francisco, California, USA.

This year’s Nobel Prize in Physiology or Medicine was awarded to three scientists who solved a decades-old mystery: how the immune system restrains self-reactive T cells that escape clonal deletion during development in the thymus. Through studies spanning the 1990s and early 2000s, Mary E. Brunkow, Fred Ramsdell, and Shimon Sakaguchi discovered and characterized regulatory T cells (Tregs) and transformed “peripheral tolerance” from a hypothetical into a proven biological system that ensures immune homeostasis (1–3). By identifying both the Treg cell itself and the transcription factor FOXP3 as the lineage’s master regulator, they opened a path to restoring immune balance specifically in diseased tissues rather than relying on broad immune system suppression with its attendant side effects. Over the past 25 years, these discoveries have reshaped our understanding of immune regulation and laid the foundation for therapies that reestablish tolerance in autoimmunity and transplantation, which may also ultimately have implications across a wide spectrum of human diseases (4).

## The people behind the breakthrough

Behind the science that led to this year’s Nobel Prize in Physiology or Medicine lies a trio of scientists whose discoveries redefined immune tolerance. Shimon Sakaguchi transformed a contentious and long-debated idea into a rigorous field, methodically uncovering the biology and mechanisms of Tregs and proving their essential role in maintaining organismal health. Drs. Brunkow and Ramsdell led the painstaking genetic sleuthing that pinpointed *FOXP3* as the gene underlying the mouse *scurfy* phenotype and human immunodysregulation, polyendocrinopathy, enteropathy, X-linked (IPEX) syndrome. Their work turned a mysterious pediatric autoimmune catastrophe into a molecularly defined disease with clear mechanistic understanding. In 2003, Ramsdell’s, Sakaguchi’s, and Alexander Rudensky’s labs linked the 2 seminal discoveries in three landmark papers that showed that the CD4^+^CD25^+^FOXP3^+^ Tregs are the quintessential guardians of peripheral immune tolerance (5–7).

This Nobel Prize also recognizes a global community that transformed foundational insights into tools, models, and medicines. Landmark advances from many laboratories around the world — spanning thymic education, peripheral tolerance, FOXP3 biology, Treg trafficking, and engineering — have matured our understanding of Tregs from a controversial cell type into a central pillar of immune tolerance. The resulting therapeutic logic is simple but profound: restore balance rather than suppress the immune system indiscriminately.

## Solving the paradox of self

T cells maturing in the thymus randomly generate many different specificities, including ones that are autoreactive. A key mechanism of immune tolerance was first revealed in the 1950s, when seminal studies showed that the majority of self-reactive T cells were deleted during T cell development in the thymus (8). This concept of “central tolerance” revealed a fundamental mechanism of immune self-restraint but did not explain how T cells that escaped thymic selection, including self-reactive T cells and T cells reactive to environmental antigens such as food and commensal microbes, were prevented from attacking healthy tissues. A specialized population of “suppressor cells,” whose primary role was to control these escapees, was proposed as the immune system’s solution to this problem (9). That idea persisted for a couple of decades but lost traction due to irreproducible experimental results and the absence of definitive molecular markers.

Nearly 25 years later, a breakthrough came from the Sakaguchi lab at the Aichi Cancer Center in Nagoya, Japan. Dr. Sakaguchi and his colleagues showed that the autoimmunity caused by neonatal thymectomy could be controlled by adoptive transfer of a small population of peripheral CD4^+^CD25^+^ cells (1). These cells, which Dr. Sakaguchi named regulatory T cells (Tregs), revived the notion that tolerance was not simply the absence of response, but an active program maintained by a small, distinct lineage of T cells with outsized control over autoimmune manifestations.

In 2001, Mary E. Brunkow and Fred Ramsdell, then at Darwin Molecular (later Celltech Chiroscience) in Seattle, were studying the genetics of catastrophic autoimmunity observed in a strain of mice bred at the Oak Ridge National Laboratory as part of a broader study of ionizing radiation on mammals. The so-called *scurfy* mouse, named for the scaly skin that developed as a consequence of multiorgan inflammation and excessive lymphoproliferation, was shown to exhibit severe autoimmune disease. Earlier studies had mapped the mutation responsible for the *scurfy* phenotype to the X chromosome. Through additional breeding and painstaking DNA mapping and sequencing approaches (remember, this took place in the late 1990s), the team pinpointed the culprit, a gene they termed *Foxp3* (2).

In the same issue of *Nature Genetics*, their team and collaborators showed that mutations in the human ortholog of the gene, *FOXP3*, cause the fatal immune dysregulation, polyendocrinopathy, enteropathy, X-linked syndrome (IPEX syndrome), which is marked by intractable diarrhea, type 1 diabetes (T1D), and eczema in young males (3). Two years later, 3 papers published in *Nature Immunology* and *Science* elegantly tied the 2 discoveries together. Drs. Rudensky, Ramsdell, and Sakaguchi demonstrated that *FOXP3* is essential for Treg development, establishing definitive markers, mechanistic clarity, and a blueprint for defining and measuring Treg biology (5–7).

In essence, Tregs evolved to prevent autoimmunity and immune dysfunction, using multiple, context-specific mechanisms to keep the immune system in balance and ensure healthy tissues and organs. These discoveries have enabled scientists to identify, expand, and manipulate Tregs — propelling exponential growth of research and the dawn of Treg-based medicines.

## Many paths, one purpose: Tregs keep the peace

The discovery of Tregs led to a fundamental shift in our understanding of immune-mediated diseases and how to treat them. For decades, clinicians managed autoimmunity and transplant rejection with nonspecific immunosuppression, using corticosteroids, cytotoxic drugs, or biologics that targeted effector cells globally but not the underlying failure of immune tolerance. We now understand that many autoimmune diseases — including T1D, lupus, rheumatoid arthritis (RA), multiple sclerosis, pemphigus, and others — are characterized by defective Treg numbers or function (10).

**Figure 1:**
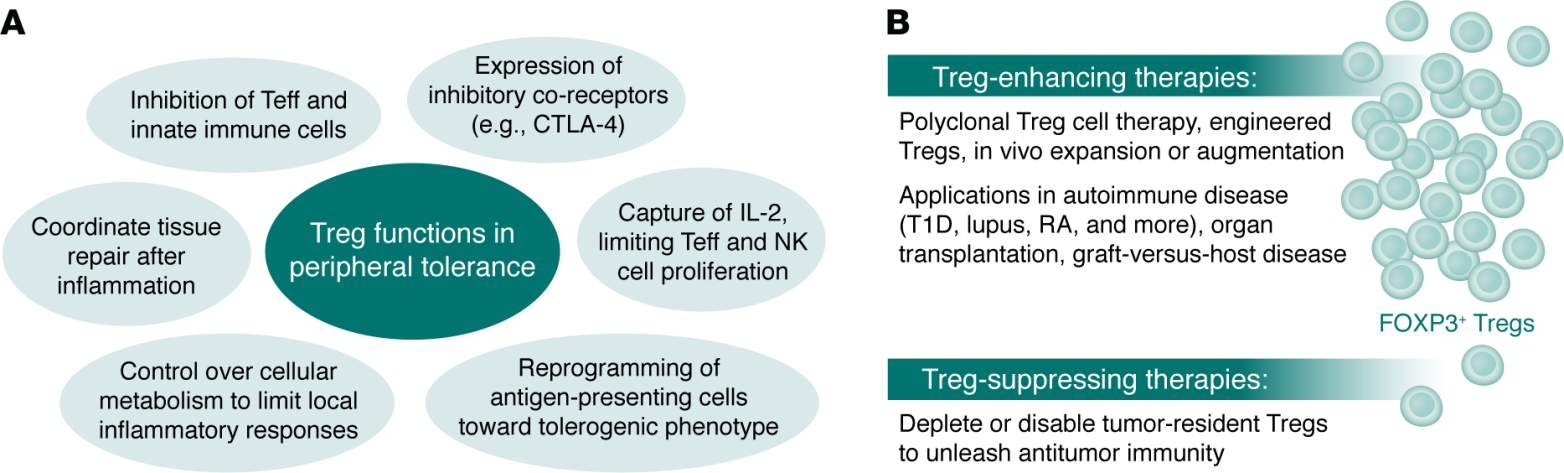
The 2025 Nobel Prize in Physiology or Medicine recognizes work by Mary E. Brunkow, Fred Ramsdell, and Shimon Sakaguchi identifying the critical functions of Tregs in maintaining central tolerance. (**A**) The relatively small population of Tregs employs diverse mechanisms to maintain tolerance and fine-tune inflammatory responses. (**B**) The discovery of Tregs formed the basis for Treg-targeted therapeutic approaches for a wide range of conditions.

The central role of Tregs in human health and disease stems from their capacity to act as a biological “polypharmacy” that is capable of fine-tuning responses with remarkable precision (Figure 1A). They deploy diverse, redundant mechanisms to quell inflammation and enforce tolerance, making them ideal candidates for therapeutic development (11). Tregs secrete immunosuppressive cytokines such as IL-10 and TGF-β, which not only directly inhibit pathogenic effector T (Teff) and inflammatory innate immune cells but also reprogram antigen-presenting cells toward a tolerogenic phenotype, thereby promoting an antiinflammatory environment. Treg expression of the IL-2 receptor α chain (CD25), which enables high-affinity capture of IL-2, allows them to act as a “sink” for this cytokine, depriving Teff and natural killer (NK) cells of a signal required for proliferation, while the expression of inhibitory co-receptors such as cytotoxic T lymphocyte–associated protein 4 (CTLA-4) alters the dynamics of costimulation needed to initiate and sustain an immune response. Tregs also limit inflammatory responses during tissue damage, converting highly proinflammatory extracellular ATP and ADP into immunosuppressive adenosine through CD39/CD73-mediated activity and controlling cellular metabolism by inducing indoleamine 2,3-dioxygenase (IDO) expression in other cells to catabolize the essential amino acid tryptophan into inhibitory kynurenines. After inflammation resolves, Tregs contribute to tissue repair by secreting growth factors and coordinating repair processes through crosstalk with local stromal, epithelial, and progenitor cells (12). This broad repertoire of suppressive and reparative mechanisms explains how a relatively small population of cells comprising just 1%–2% of T cells in blood and lymphoid tissues can shut down complex inflammatory responses with precision and efficiency and highlights the therapeutic potential of harnessing these functions to restore immune equilibrium. Finally, Tregs amplify their activity through a process termed infectious tolerance, which can turn other cells into regulatory cells locally in inflamed tissues (13).

## From discovery to therapy: restoring immune balance

The realization of the polypharmacologic activity and potency of Tregs has led to two complementary strategies on how to apply these functionalities in the emerging field of tolerance medicine: (a) the use of cell therapeutics using polyclonal Treg cell therapy (14), and, more recently, engineered Tregs (15), in which a patient’s Tregs are isolated, transduced with disease-homing or antigen-specific receptors, expanded ex vivo, then reinfused; and (b) in vivo augmentation of Tregs using low-dose IL-2 (16), engineered IL-2 variants (17), TNFR2 agonists, tolerogenic autoantigen peptides, and other biologics that selectively boost Tregs and restore balance (18) (Figure 1B). These approaches have entered early clinical trials in autoimmunity, organ transplantation, and the prevention/treatment of graft-versus-host disease, with second-generation products now refining Treg homing, stability, and function (19).

Moreover, it has become clear that inflammation is a key driver even of diseases and normal physiology not classically considered immune mediated. From metabolic (20) and cardiovascular disorders (21) to neurodegeneration (22), muscular dystrophy (23), and even obesity (24), pregnancy/reproductive diseases (25) and aging (26). In addition, tissue-resident Treg dysfunction is involved in chronic inflammation that contributes to a wide range of degenerative conditions often associated with it. Recognition of inflammation’s prevalent role in disease pathogenesis places Treg therapies at the crossroads of human health and underscores the untapped potential of harnessing these powerful immune regulators to treat chronic diseases.

Finally, in opposition to the role of Tregs in autoimmunity, we now appreciate that an excess of Tregs can suppress antitumor responses, shielding tumors from immune attack and worsening prognosis. Insights from Treg biology have clarified the mechanisms underlying checkpoint inhibitors such as anti–CTLA-4 and other checkpoints, which can result in serious and clinically significant autoimmune side effects, and have inspired next-generation therapies. New therapies are being developed that can be applied in reverse to selectively deplete or disable tumor-resident Tregs to unleash antitumor immunity, including selective depletion of highly suppressive CCR8^+^ Tregs within tumors (27). This places Tregs firmly at the center of both autoimmunity and protective immunity.

## At the clinical frontier: promise and practical challenges

By revealing Tregs and uncovering FOXP3 as their master regulator, Brunkow, Ramsdell, and Sakaguchi provided the conceptual and molecular levers to control the immune system with unprecedented finesse. Their work explains why, in many cases, autoimmunity arises, how tolerance is actively maintained, and how we might reestablish that balance when it fails. Three decades on, these insights are no longer just elegant biology — they form a translational playbook guiding the development of cell therapies, biologics, and combination approaches now entering the clinic.

The field of tolerance medicine is at an inflection point, where biology and technology are converging to mobilize Tregs as precision therapeutics and create new opportunities for clinical translation. Treg isolation protocols have advanced substantially with the identification of lineage-specific markers that allow their isolation at scale without effector cell contamination, enabling expansion of the small number of Tregs obtained from a patient into a therapeutic dose. In some cases, the *FOXP3* gene itself is introduced into bone marrow (28), naive or Teff cells with the goal of transforming them into Tregs. In addition, efforts are underway to enhance Treg survival and persistence, such as cytokine supplementation and transient Teff cell depletion. But we are still at the beginning of this burgeoning field. The future may include off-the-shelf Treg therapies, direct manipulation of Tregs in vivo using gene therapy to introduce CARs and other genes into Tregs (not unlike what is being pioneered in the CAR-T effector cell therapies), and fine-tuning for disease indications.

The field-defining work of Brunkow, Ramsdell, and Sakaguchi has inspired a new generation of scientists to take up the mantle, and their ongoing research may ultimately move Treg therapies from therapeutic possibility to disease-changing drugs. This Nobel Prize celebrates not only a decisive scientific arc from hypothesis to mechanism to medicine but also a therapeutic promise: the precision restoration of immune balance for the patients who need it most.

## Version 1 12/01/2025

Electronic publication
